# Evaluating the precision and accuracy of digital dental models with a low-cost structured light device

**DOI:** 10.1590/2177-6709.29.1.e2423217.oar

**Published:** 2024-03-29

**Authors:** Vitor de Toledo STUANI, Matheus Dante de PAULA, Raphaella Coelho MICHEL, Gustavo Gonçalves do Prado MANFREDI, Edna Maria de Oliveira FERNANDES, Diana Gabriela Soares dos PASSOS

**Affiliations:** 1 Universidade de São Paulo, Faculdade de Odontologia de Bauru, Departamento de Dentística, Endodontia e Materiais Odontológicos (Bauru/SP, Brazil).; 2 Universidade de São Paulo, Hospital de Reabilitação de Anomalias Craniofaciais (Bauru/SP, Brazil).; 3 Universidade de Saúde e Ecologia Humana, Faculdade de Odontologia, Disciplina de Periodontia (Vespasiano/MG, Brazil).; 4 Universidade Estadual do Norte do Paraná, Disciplina de Implantodontia (Jacarezinho/PR, Brazil).

**Keywords:** Dental informatics, Three-dimensional image, Data accuracy, Informática odontológica, Imagem tridimensional, Precisão de dados

## Abstract

**Objective:**

This study evaluated the accuracy and precision of digital models acquisition using a home-built, low-cost scanning system based on the structured light method.

**Methods:**

a plaster model (PM) was scanned using the experimental device (SL) and a dental desktop scanner (DS). The teeth dimensions of PM and SL models were measured in triplicate, with a caliper and digitally, respectively. The agreement of the measurements of each model was evaluated using the intraclass correlation coefficient, and the validity between the different measurement techniques was assessed using the Bland-Altman analysis. The accuracy and precision of the models were qualitatively investigated using the mesh superposition of the SL and DS models.

**Results:**

A high intraclass correlation coefficient was observed in all models (PM=0.964; SL1=0.998; SL2=0.995; SL3=0.998), and there was no statistical difference between the measurements of the SL models (*p*>0.05). PM and SL model measurements were found to be in good agreement, with only 3.57% of the observed differences between the same measurement being located outside 95% limits of agreement according to Bland and Altman (0.43 and -0.40 mm). In the superimpositions of SL-SL and SL-DS models, areas of discrepancy greater than 0.5 mm were observed mainly in interproximal, occlusal, and cervical sites.

**Conclusion:**

These results indicate that the home-built SL scanning system did not possess sufficient accuracy and precision for many clinical applications. However, the consistency in preserving the dental proportions suggests that the equipment can be used for planning, storage, and simple clinical purposes.

## RESUMO

**Objetivo:**

Este estudo avaliou a acurácia e a precisão da aquisição de modelos digitais utilizando um sistema de digitalização caseiro e de baixo custo baseado no método de luz estruturada.

**Material e Métodos:**

Para isso, um modelo de gesso (MG) foi digitalizado utilizando o dispositivo experimental (LE) e um *scanner* dental de mesa (SD). As dimensões dos dentes dos modelos MG e LE foram medidas em triplicata com um paquímetro e digitalmente, respectivamente. A concordância das medidas de cada modelo foi avaliada usando o coeficiente de correlação intraclasse, e a validade entre as diferentes técnicas de medição foi avaliada usando a análise de Bland-Altman. A acurácia e a precisão dos modelos foram investigadas qualitativamente usando a sobreposição de malhas dos modelos LE e SD.

**Resultados:**

Um alto coeficiente de correlação intraclasse foi observado em todos os modelos (MG=0,964; LE1=0,998; LE2=0,995; LE3=0,998) e não houve diferença estatística entre as medições dos modelos LE (*p*>0,05). As medições dos modelos MG e LE mostraram boa concordância, com apenas 3,57% das diferenças observadas entre as mesmas medições localizadas fora dos limites de concordância de 95% de acordo com a análise de Bland-Altman (0,43 e -0,40 mm). Nas sobreposições dos modelos LE-LE e LE-SD, foram observadas áreas de discrepância maiores que 0,5 mm principalmente nos sítios interproximais, oclusais e cervicais.

**Conclusões:**

Esses resultados indicam que o sistema de digitalização por luz estruturada caseiro não possui precisão e acurácia suficientes para muitas aplicações clínicas. No entanto, a consistência na preservação das proporções dentárias sugere que o equipamento pode ser usado para planejamento, armazenamento e propósitos clínicos simples.

## INTRODUCTION

In recent years, a paradigm shift has marked the evolution of dentistry. The refinement of CAD/CAM (Computer-Aided Design / Computer-Aided Manufacturing) systems has intensified the migration of clinical activity towards a cyber-physical environment, optimizing the treatment planning and execution.^1^ ^,^ ^2^ However, it is of major importance to highlight the high investment required for the adoption of these new technologies. This leads to an excluding scenario for many practitioners in developing countries, making these technologies inaccessible to underserved populations. For this reason, it is of utmost importance to evaluate possible alternatives that can reduce costs and facilitate the diffusion of digital dentistry communally.

In most cases, the digital workflow in dentistry begins with the digitalization of teeth and oral structures. This process can be done directly or indirectly, with the latter using molds or plaster models obtained from the patient. For this, computed tomography, laser scanning, photogrammetry, Moiré topography, and structured light techniques can be used.^1^ ^-^ ^4^ Among these alternatives, structured light has shown advantages regarding its high resolution and capture frequency, although it is prone to the presence of noise.^1^ Another advantage is that structured light offers no risk of tissue damage, allowing the scanning of individuals in complete safety.^5^ For these reasons, this technique has been widely spread as accurate, fast, safe, and versatile.^6^

The structured light scanning technology is based on a non-contact active scanning method where the surface reconstruction is done through a triangulation process. The process begins with a projector emitting a pattern of structured light onto an object, causing the projected lines to deform over its surface. These patterns are captured by cameras, allowing algorithms to perform the three-dimensional reconstruction of the object.^6^ ^,^ ^7^ The use of this technique with professional and non-dental equipment has already demonstrated satisfactory results for clinical use in some situations.^7^ ^-^ ^10^ When comparing the discrepancy between the measurement of physical models and digital models obtained using structured light systems, DeLong et al.^11^ and Del Corso et al.^12^ observed values of 18-30 µm and 14-21 µm, respectively. These values are within the range observed in dental scanners, where accuracy values between 7.7 and 46 µm and precision values between 4 and 50 µm can be obtained.^13^ ^-^ ^20^

With the present evidence regarding the potential of using structured light in dentistry, added to the reduced cost of the components of this system, the use of this technology can serve as a gateway to the digital workflow for professionals based on economically compromised areas. With this, underserved populations would also have access to treatments with greater quality, predictability, and comfort. Thus, this pilot study aimed to evaluate the accuracy and precision of a low-cost, home-built structured light system for digitizing dental models. This apparatus was assembled by our group through the substitution of dedicated industrial scanning equipment with low-cost components, enabling its compatibility with the selected software. The hypothesis was that there would be no significant differences between measurements taken on a dental model using a digital caliper and those obtained on digital models reconstructed using the structured light device. The null hypothesis was that there would be no correspondence between the methods.

## MATERIAL AND METHODS

### DIGITAL MODEL ACQUISITION

**Figure 1:**
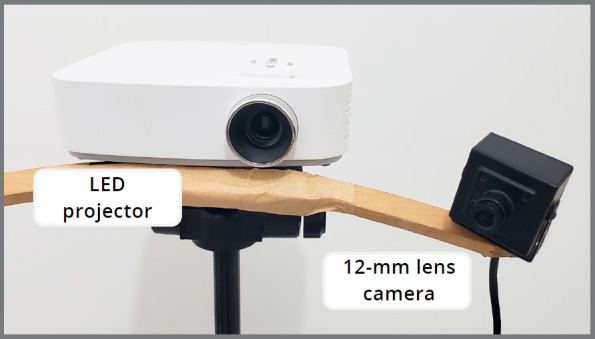
Home-built, cost-effective structured light scanning setup. Light patterns were projected using a LED projector (PF50KS, LG Corporation, Seoul, South Korea), and images were captured by a high-resolution 12-mm lens camera (ELP 1080P HD, Ailipu Technology Co., Ltd, Shenzhen, China).

A home-built, low-cost structured light scanning system was set up using an LED projector (PF50KS, LG Corporation, Seoul, South Korea) with a native resolution of 1,920 x 1,080 pixels and brightness of 600 ANSI lumens. For image capturing, a high-resolution, 12-mm lens camera (ELP 1080P HD, Ailipu Technology Co.,Ltd, Shenzhen, China) was attached laterally to the projector (Fig 1). This set was stabilized on a tripod and positioned at a focal length of about 40 cm from the scanning area. The scanning was performed using a plaster replica of a dental model (P-Oclusal Prod. Odont. Ltda., São Paulo/SP, Brazil), since plastic products with reflective surfaces can impair image capture. The plaster model (PM) was kept on a rotating platform, and a new capture was performed at every 15° of rotation. The scanning was performed using a DAVID 5 3D Scanner software (Hewlett-Packard Company, Palo Alto, USA) after calibrating the equipment. The calibration followed the manufacturer recommendations. Briefly, a 90° pair of glass panels with known dimensions was used as a reference. The calibration corner was set up by placing fixing brackets on a flat surface and inserting the glass panels. The calibration pattern was chosen based on the object size. The glass panels and scanner were positioned for optimal projection and camera image clarity, ensuring visibility of calibration markers. The correct scale length was entered, and projector brightness was adjusted. The scanner was calibrated for position, rotation, focus, and brightness. The entire procedure was conducted in a dark environment, with windows and doors sealed, to prevent any entry of light. Three scans were performed by the same operator, resulting in three digital models (SL1, SL2, and SL3). The generated STL files were imported into NemoStudio planning software (Nemotec, Madrid, Spain) to orient their coordinates and finishing of the three-dimensional mesh.

### MODEL MEASUREMENT

**Figure 2:**
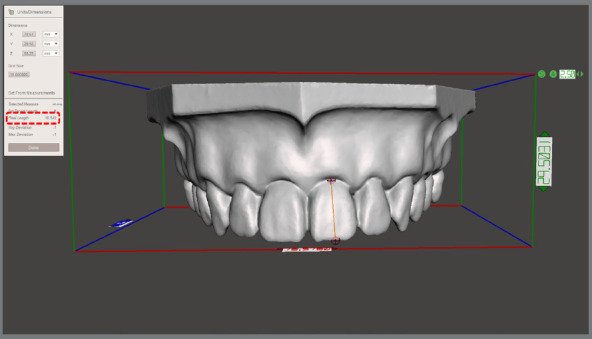
Demonstration of the measurement methodology employed to assess the height and width dimensions of teeth in the digital models obtained using the structured light device. Measurements were conducted using the Meshmixer software (Autodesk Inc., San Rafael, CA, USA) with the Units/Dimension tool.

To evaluate the reproducibility of dental dimensions, the heights and widths of each tooth on the plaster model (PM) were measured using a digital caliper, keeping the measurement of two decimal digits. The digital models obtained with the structured light scanning (SL) had the same measurements obtained using the Meshmixer software (Autodesk Inc., San Rafael, CA, USA) with the tool Units/Dimension (Fig 2). All measurements were performed in triplicate by a single calibrated evaluator, and the correlation coefficient of measurements was calculated after each process.

### MESH SUPERIMPOSITION

The accuracy of the structured light scanning device was qualitatively evaluated through the superposition of the SL models. In turn, its precision was verified through the superposition of the SL models and a digital model (DS) obtained through the scanning of the plaster model using a desktop scanner (R700™ scanner; 3Shape A/S, Copenhagen, Denmark). The generated STL file was imported into NemoStudio planning software (Nemotec, Madrid, Spain) for three-dimensional repositioning and finishing of the three-dimensional mesh. The superimposition of images was conducted utilizing GOM Inspect 2018 software (GOM Metrology, Braunschweig, Germany), with the pre-alignment tool - this tool ensures a globally optimal fit, by minimizing all deviations between the meshes while adjusting their spatial coordinates.

### STATISTICS

All collected data were recorded in a Microsoft Excel spreadsheet (Microsoft Corporation, Redmond, WA, USA), and the Shapiro-Wilk test was applied to assess the normality of their distribution (SPSS Statistics; IBM Corpate, Armonk, NY, USA). The agreement between the measurements obtained in models SL1, SL2 and SL3 was evaluated by repeated measures ANOVA *post-hoc* Tukey test, using the Jamovi software.^21^ The degree of agreement between the measurements of the PM and SL models was evaluated using the Bland-Altman method with SPSS Statistics software (IBM Corpate, Armonk, NY, USA). All measurements were obtained in triplicate on different days, and the examiner correlation coefficient analysis was performed using the Jamovi software^21^ (Jamovi, Sydney, Australia). For all analyses, it was adopted a significance level of 5% and a power of >80%.

## RESULTS

**Figure 3:**
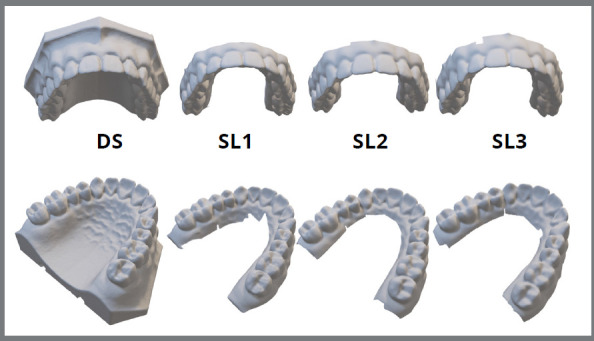
Scanned models obtained using desktop scanning (DS) and using structured light (SL1-SL3).

All models obtained using structured light scanning showed visual quality and finishing similar to desktop scanning (Fig 3). The values found in the measurements of the plaster model (PM) and the structured light scannings (SL) are presented in the Table 1. The evaluator’s concordance correlation showed high values for repeated measurements (PM=0.964; SL1=0.998; SL2=0.995; SL3=0.998). Furthermore, the repeated measures analysis indicated no statistical difference between the measurements of models SL1, SL2 and SL3 (Table 2).

**Table 1:** Data obtained in the measurement of the SL and PM models, indicating a normal distribution in the groups (Shapiro-Wilk, p<0.05).

TOOTH	PM		SL1		SL2		SL3	
	Height	Width	Height	Width	Height	Width	Height	Width
27	8.01±0.12	9.47±0.35	8.16±0.05	9.30±0.07	7.90±0.09	9.20±0.05	7.86±0.05	9.42±0.03
26	7.67±0.22	9.94±0.31	7.86±0.06	10.11±0.17	7.17±0.03	9.93±0.06	7.17±0.04	9.85±0.12
25	8.41±0.25	5.74±0.13	8.17±0.09	6.01±0.02	8.26±0.01	6.17±0.09	8.16±0.06	6.16±0.02
24	9.49±0.26	6.85±0.11	9.59±0.11	6.80±0.02	9.45±0.05	6.70±0.1	9.21±0.05	6.72±0.01
23	10.35±0.25	7.27±0.16	10.26±0.04	6.85±0.12	10.22±0.07	7.05±0.07	10.23±0.02	7.18±0.13
22	10.01±0.03	7.39±0.04	10.01±0.06	7.62±0.16	10.01±0.13	7.13±0.09	9.96±0.07	7.04±0.06
21	10.55±0.15	8.76±0.2	10.28±0.06	8.82±0.08	10.17±0.07	8.70±0.2	10.31±0.03	8.63±0.07
11	10.52±0.14	8.44±0.38	10.49±0.04	8.94±0.03	10.34±0.07	8.88±0.04	10.32±0.04	8.90±0.06
12	9.51±0.16	6.52±0.46	9.29±0.04	6.84±0.02	9.19±0.04	6.85±0.06	9.10±0.07	6.80±0.04
13	10.42±0.19	7.24±0.11	10.28±0.07	7.44±0.08	10.20±0.02	7.42±0.04	10.26±0.07	7.38±0.06
14	9.02±0.42	6.41±0.47	8.81±0.07	6.43±0.08	8.68±0.05	6.56±0.12	8.62±0.07	6.63±0.12
15	8.00±0.1	5.78±0.17	7.97±0.05	5.97±0.14	7.81±0.03	6.06±0.16	7.45±0.06	6.04±0.06
16	7.78±0.12	9.8±0.51	7.82±0.06	10.16±0.06	7.85±0.03	10.25±0.1	7.82±0.05	10.23±0.04
17	7.33±0.21	9.11±0.18	7.46±0.04	8.71±1.1	7.45±0.04	8.79±0.08	7.43±0.01	8.90±0.04
Shapiro-Wilk p	0.211	0.255	0.128	0.194	0.070	0.157	0.062	0.147

**Table 2:** Comparison between the measurements collected in the SL models (repeated measures ANOVA *post-hoc* Tukey, p<0.05).

Comparison		Mean Difference	SE	df	t	p Tukey
Digital Model	Digital Model					
SL1	SL2	0.0739	0.0352	27.0	2.10	0.108
	SL3	0.0950	0.0448	27.0	2.12	0.105
SL2	SL3	0.0211	0.0210	27.0	1.00	0.582

**Figure 4:**
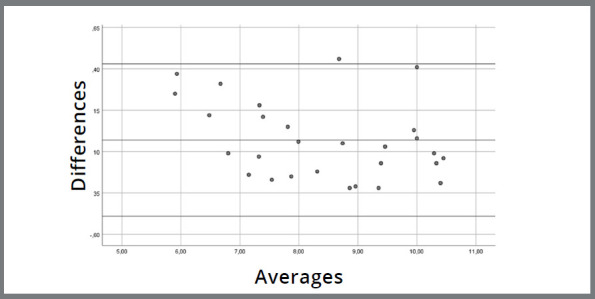
Evaluation of the accuracy of SL, compared to PM measurements. The central line represents the Mean of the difference between analog and digital measurement methods at each evaluated site (14 height and 14 width measures). The upper and lower lines correspond to a 95% confidence interval, determined by the Mean of the differences ± (1.96 x Standard Deviation of the differences). The proximity of the central line to zero indicates agreement between the values obtained from both measurement methods. Except for one site, all others fall within the confidence interval, suggesting a relatively good agreement between the methods.

As for the comparison of the PM and SL model measures, the one-sample *t*-test of the differences between the groups’ means did not indicate a significant difference from 0 [Sig. (2-tailed) = 0.452]. This suggests an agreement between the two methods, since the measures are so similar that their differences do not differ from 0 (Fig 4). Except for one measurement, all of them can be observed within the 95% confidence interval (0.43 and -0.40 mm).

Additionally, a linear regression model was performed to assess the existence of a proportion bias between the measurement differences. The result indicated the absence of bias (*p*=0.07), suggesting that the proportion was homogeneously distributed between the mean differences of the two methods.

**Figure 5:**
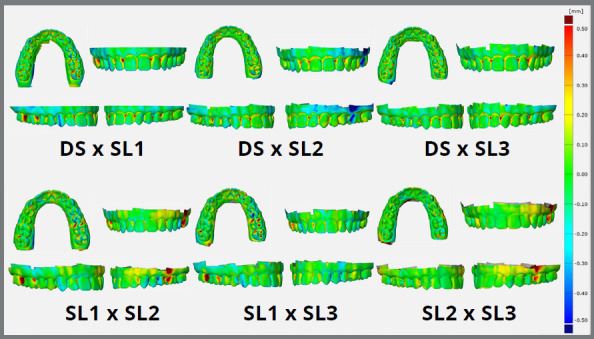
Superimposition of SL (Structured Light) and DS (Digital Scan) models for qualitative assessment of the accuracy (PM vs. SL1-3) and precision (SL1 vs. SL2 vs. SL3) of the custom-built structured light device. The color map ranges from -0.5 mm (dark blue) to +0.5 mm (red). Negative discrepancies (from sky blue to dark blue) signify a smaller crown compared to the master model, while positive discrepancies (from yellow to red) indicate a larger crown than the master model. Green represents an agreement within the range of +0.1 mm to -0.1 mm.

Similarly, the superimposition of the DS and SL digital models indicates acceptable accuracy, with a positive or negative discrepancy range of 0 - 0.5 mm (Fig 5). The same was observed in the accuracy analysis, with the SL models superimposed on each other. The difference between the models was more pronounced in the interproximal, cervical and occlusal sites, indicating a possible limitation in capturing the light in these regions. Thus, the use of structured light was able to replicate the model in great detail. However, the distortions observed make the models inadequate for a variety of clinical applications.

## DISCUSSION

The objective of this pilot study was to evaluate the accuracy and precision of a low-cost structured light scanning system as an alternative for digitizing dental models. This effort focused primarily on providing a gateway to a digital workflow for dental surgeons who do not have access to high-cost technological resources and who work with socially underprivileged groups. As the focus of this study was to promote the accessibility to digital dentistry, it was decided to use a home-built system with low-cost hardware and software, when compared to traditional dental scanners. 

Structured light scanning technology is based on the projection of a known pattern of light over the object of interest. This pattern deforms itself along the scanned surface, and these perturbations identify the geometry of the object. Then, the reconstruction algorithm of the software identifies the spatial positioning of millions of points captured by the camera, and digitally reconstructs the object. The principle of this technique is based on concepts of classical photogrammetry, ensuring accuracy and precision compatible with various applications, such as reverse engineering and biometrics.^22^ One of the reasons that motivated the adoption of a structured light system device in this pilot study was the possibility of simplifying its physical components with the use of high-resolution projectors and cameras, and the diversity of software that could be used at the reconstruction stage. Although the use of non-specific equipment can affect the quality of the scan, the development of a home-built structured light system facilitates the customization of the scanner parameters and results in a significant reduction in overall cost.

It is noteworthy that all models obtained by structured light scanning presented visual quality and finishing similar to that obtained with a dental desktop scanner. Furthermore, an agreement was observed between the teeth heights and widths measured in the digital (SL) and plaster (PM) models. To evaluate the reliability of these measurements, it was used the intraclass correlation coefficient (ICC), which was first described by Barkto.^23^ In this pilot study, we observed a slightly higher value in the ICC of SL models. This may be a reflection of the measurement technique itself, since the handling of the digital caliper may have contributed to a rougher difference between measurements of PM. However, it should be noted that all evaluations showed excellent reliability (PM=0.964; SL1=0.998; SL2=0.995; SL3=0.998).^24^ Liu et al.^9^ obtained similar ICC values when evaluating measurements performed on physical models and models obtained with a dental structured light scanner. For mesiodistal measurements on physical models, they obtained an ICC of 0.968-0.984 for maxillary teeth. On the digital models, this value was 0.965-0.989. When assessing crown height, however, a more marked difference was found, with the ICC for upper teeth on the physical models being 0.959-0.970, and 0.985-0.999 on the digitals. Unlike the methodology used by these authors, the ICC was not stratified between vertical and horizontal measurements in the present study, which may have accentuated the overall difference at each measurement technique.

To verify the agreement between the data obtained from the measurement of the physical and digital models, it was used the analysis proposed by Bland and Altman.^25^ This evaluation investigates the validity of the same quantitative variable measured by two different techniques. In this pilot study, except for one measurement, the differences in the means of all measurements were within the 95% confidence limit (0.43 and -0.40 mm). This means that only 3.57% of the measurements were outside the confidence limits, which is similar to the results observed by Liu et al.^9^ for the maxilla (5.20-5.43% in mesiodistal dimension, and 4.71-5.29% in height). Moreover, all the differences recorded in our study had a value lower than ±0.5 mm, which is considered clinically acceptable by some authors.^10^ ^,^ ^26^ These results are in agreement with those reported by other groups that compared physical and digital measurements using models obtained by different scanning processes.^9^ ^,^ ^10^ ^,^ ^27^ ^-^ ^32^ Thus, it is possible to say that the home-built structured light scanner was able to generate models whose teeth presented mesiodistal and height measurements with clinically acceptable precision and accuracy.

These results suggest the beneficial applicability of this technique in the digitization of models for diagnostic, prosthetic and orthodontic studies, or for storage of patient records. Plaster models require substantial storage space over a variable period, according to the recommendations of each country’s regulatory agency. In addition, retrieving a model in a physical archive can be laborious and they can be easily damaged. For this reason, the use of digitization tools that allow models to be stored on hard disks or in the cloud provides great convenience, organizational optimization, spatial availability, resource savings, reusability and repeatability of analyses, and allows professionals in remote locations to work cooperatively with colleagues around the world.

Although the agreement between the physical and digital model measurements was promising, the superimposition of the SL and DS models indicated areas with less than the recommended accuracy for some clinical applications. Overall, extraoral scanning can be performed using laser, structured light, or contact equipment. Although optical scanners (laser and structured light) are not influenced by the object density, they are affected by the optical properties of the scanned target, the environment, the scanning strategies, and the data processing.^33^ Thus, the interaction of these factors may have led to areas of increased discrepancy between models.

In regards to structured light scanning, it must be highlighted that a high accuracy relies on proper calibration of the equipment. The purpose of calibration is to provide reference data to the reconstruction software so that it comprehends the spatial arrangement of the light-emitting source, the camera, and the scanning area. In this pilot study, calibration was performed using a printed grid of known 2D patterns adhered to a rigid plate. Thus, the printing process may have induced minor dimensional variations in the grid and thus affected the renderization of SL models.^22^ Furthermore, any surface deformity of the paper adhered to the plate may have contributed to an increase in reprojection error, since the reconstruction software estimates that this surface is completely flat.^22^

Despite the precautions taken to perform SL scans consecutively and in the same dark room, it is impossible to say that no environmental modification occurred during the process. Thus, variations in temperature and light may have partially contributed to the differences between the scanned models. Ambient light has a great impact on the quality of the digital file. An external light source can change the saturation and intensity of the light captured by the camera, leading to a calculation error.^22^ In addition, variations in ambient temperature can alter the quality of the scan due to a lack of mechanical stability of the object.^22^

Overall, it was possible to observe a considerable accuracy in the superimposition of meshes, with a positive or negative discrepancy of 0.5 mm delimited to certain sites. Areas of lower accuracy and precision were mostly located in the interproximal surfaces, and a discrepancy was also noted in cervical and occlusal sites. This deviation can be explained by the inability of the light to illuminate and decode the patterns reflected in these regions, as concave surfaces are challenging for scanners with large triangulation angles.^22^ Therefore, scanners are more accurate on smooth surfaces and perform inferiorly in occlusal grooves or cervical areas.^19^ This is particularly alarming when considering the scanning of a finishing line of tooth preparation since a gap of more than 120 µm is not considered clinically acceptable.^34^

On the other hand, it is relevant to mention that dental scanners present a large variation in accuracy according to the technique and equipment used, making this low-cost equipment competitive in some cases. Bohner et al.^33^ reported accuracy in dental scanners of 55-116 µm in full arches, and up to 698 µm in edentulous models. In addition, the use of cone-beam computed tomography scans has resulted in a widely varying accuracy, ranging from 106 to 760 µm. Thus, although considerably inferior to dental scanners, the home-built structured light system can be used in specific situations, as long as its limitations are known.

Therefore, its use is not recommended for the production of definitive prostheses, given the deficiency observed in the interproximal, cervical and occlusal regions. On the other hand, provisional prostheses could be made, as long as a reline is made to repair crown adaptation. Furthermore, a greater occlusal adjustment and more extensive care in establishing the contact point will be necessary, when compared to the use of other scanning techniques. Also, the use of this device is not recommended for planning high-precision procedures, such as surgical guides in areas surrounded by noble anatomical structures. Conversely, further studies should be performed to evaluate the predictability of using it to make surgical guides for single implants in areas of high bone availability.

Although it was not the scope of this pilot study, it is also possible to speculate that the device could be used in facial scans to assist in the diagnostic, planning, and implementation of dental and medical treatments. According to a recent systematic review,^33^ facial scanners have an average accuracy of 500 µm, with a discrepancy of up to 2 mm being considered clinically acceptable. Further studies should be conducted to verify this possibility.

Finally, another limitation of this study lies in the choice of the DAVID 5 3D Scanner software (Hewlett-Packard Company, Palo Alto, USA), which has been discontinued, posing challenges to its usability. Nevertheless, the same methodology can be applied to similar software alternatives, with options such as the FlexScan3D (Polyga, Vancouver, Canada) offering monthly subscription plans, or free open-source alternatives like SLStudio (Institute of Electrical and Electronics Engineers, Washington, USA) and 3D Underworld (FP7 Marie Curie Fellowship, Montréal, Canada). However, it will be necessary to overcome a significant learning curve to become familiar with this technology.

## CONCLUSION

The home-built structured light scanning device effectively reproduces vertical and horizontal dimensions of teeth in a full arch model. This allows for creating digital databases, streamlining document preservation and information exchange with dentists globally. Storing data on hard disks or in the cloud eliminates the need for physical archiving spaces, reducing operational costs. While the scanner may not be precise enough for certain procedures like definitive prostheses or surgical guides, its affordability makes it a potential entry point for dentists in economically disadvantaged areas, provided they are aware of its limitations. Further preclinical research is needed to assess the technique’s predictability in various clinical settings.
